# The Hippo signaling pathway: a potential therapeutic target is reversed by a Chinese patent drug in rats with diabetic retinopathy

**DOI:** 10.1186/s12906-017-1678-3

**Published:** 2017-04-04

**Authors:** Gai-mei Hao, Tian-tian Lv, Yan Wu, Hong-liang Wang, Wei Xing, Yong Wang, Chun Li, Zi-jian Zhang, Zheng-lin Wang, Wei Wang, Jing Han

**Affiliations:** 1 College of Basic Medicine, Key Laboratory of Ministry of Education (Syndromes and formulas), Key Laboratory of Beijing (Syndromes and formulas), Beijing University of Chinese medicine, Beijing, China; 2 Institute of Chinese Medicine, Beijing University of Chinese medicine, Beijing, China; 3 Institute of Basic Theory for Chinese Medicine, China Academy of Chinese Medical Sciences, Beijing, China; 4 Modern Research Center for Traditional Chinese Medicine, Beijing University of Chinese Medicine, Beijing, China

**Keywords:** Diabetic retinopathy, Chinese medicine, Hippo

## Abstract

**Background:**

The Hippo signaling pathway is reported to be involved in angiogenesis, but the roles of the Hippo pathway in diabetic retinopathy have not been addressed. Fufang Xueshuantong Capsule has been used to treat diabetic retinopathy in China; however, the effect of Fufang Xueshuantong Capsule on the Hippo pathway has not been investigated.

**Methods:**

In this study, diabetes was induced in Sprague-Dawley rats with intraperitoneal injection of streptozotocin. Twenty weeks later, Fufang Xueshuantong Capsule was administered for 12 weeks. When the administration ended, the eyes were isolated for western blot and immunohistochemistry analyses. The levels of P- mammalian sterile 20-like (MST), large tumor suppressor homolog (Lats), P- yes-associated protein (YAP), transcriptional co-activator with PDZ binding motif (TAZ) and TEA domain family members (TEAD) were measured.

**Results:**

Diabetic rats had a decreased P-MST level in the inner plexiform layer and reduced expression of P-YAP in the photoreceptor layers of their eyes. In addition, diabetic rats displayed remarkable increases in Lats, TAZ and TEAD in their retinas. Furthermore, Fufang Xueshuantong Capsule restored the changes in the Hippo pathway.

**Conclusions:**

The Hippo signaling pathway is important for the progression of diabetic retinopathy and will hopefully be a targeted therapeutic approach for the prevention of diabetic retinopathy.

**Electronic supplementary material:**

The online version of this article (doi:10.1186/s12906-017-1678-3) contains supplementary material, which is available to authorized users.

## Background

Diabetic retinopathy (DR) is one of the most prominent complications of diabetes. DR remains a leading cause of blindness and is characterized by vascular abnormalities, including increased permeability and the growth of new blood vessels [1]. At present, controlled systemic deregulation, laser photocoagulation, vitreoretinal surgery and intravitreal anti-vascular endothelial growth factor (VEGF) drugs remain the most common approaches to slow the development and progression of DR [2]; however, some existing limitations and the fact that some patients respond poorly to these treatments has led to the need for novel therapies for DR [3]. In addition, these findings suggest suggests that some other mediators or pathways may participate and play an important role in DR. Therefore, it is important to elucidate the exact mechanisms of DR and then investigate targeted ways to arrest the progression of this disease.

The Hippo pathway is newly discovered and plays important roles in organ size control [4]. The pathway consists of a large number of kinases and proteins, and the core members are mammalian sterile 20-like(MST), large tumor suppressor homolog(Lats), MOB kinase activator1(Mob1), and salvador1(hsav1) [5]. The kinase cascade phosphorylates yes-associated protein(YAP), transcriptional co-activator with PDZ binding motif(TAZ) and TEA domain family members(TEAD), which control cell proliferation [6, 7].

It has been reported that Lats [8] and YAP [9] participate in the proliferation of endothelioma cells and vascular smooth muscle cell, respectively [10, 11]. In addition, Lats [8] and YAP [10] regulate angiogenesis in zebrafish. It is striking that YAP, which is the effector of the Hippo pathway, is expressed in retinal vessels and involved in endothelial sprouting [12] and angiogenesis [13]. It is well known that angiogenesis, which accompanies the proliferation of endothelial cells, is a symptom of DR. Thus, these previous studies suggest that the Hippo pathway plays roles in DR.

To identify the specific roles of the Hippo signaling pathway in the progression of DR, the influence of Fufang Xuesuhangtong (XST) Capsule, a patented Chinese drug that is reported to impede DR, on the Hippo pathway was investigated. XST, which is composed of *Panax notoginseng*, *Salvia miltiorrhiza*, *Astragalus membranaceus* and *Scrophularia ningpoensis*, has been noted for its medicinal effect against DR for almost 20 years. Many clinical studies have indicated that XST could prevent fundus hemorrhage and exudation and stable vision in DR patients [14]. Afterwards, XST has been found to ameliorate whole blood viscosity, plasma viscosity, and erythrocyte aggregation indexes in STZ-induced rats [15]. Moreover, XST suppresses the acellular capillaries and increase the pericyte numbers, which are the characteristics of DR in rats [15]. In addition XST decreases the basement membrane thickness of the capillary and improved the pathological changes of the ganglion cells in the retina of diabetic rats [16]. Meanwhile XST attenuates the aldose reductase activity and the content of malondialdehyde (MDA), diminishes the expression of VEGF, intercellular cell adhesion molecule-1 (ICAM-1) and inducible nitric oxide synthase (iNOS), and augments the level of superoxide dismutase (SOD), pigment epithelium-derived factor (PEDF) and occludin [15, 16]. Furthermore, recent studies have shown that the characteristic constituents of XST have the same pharmacological effect on DR [17].

We hypothesize that the Hippo signaling pathway participates in the process of DR and that XST has an impact on this pathway. Thus, the expression or distribution of proteins in the Hippo pathway was investigated in the retinas of diabetic rats. In addition, the changes in the core members in the Hippo pathway were examined in rat retinas after XST administration. This study will enhance the understanding of the mechanisms promoting the development of DR and provide valuable indications for a novel therapeutic target for DR.

## Methods

### Ethics statement

All procedures involving animals and their care were carried out according to the governmental guidelines on animal experimentation and the National Institutes of Health’s “Principles of Laboratory Animal Care”. All experimental protocols were approved by the Institutional Animal Ethics Committee of Beijing University of Traditional Chinese Medicine, Beijing, China (Permit Number: 26–1514).

### Antibodies

Anti-VEGF antibody, Abcam (Cambridge, UK), ab1316, mouse monoclonal, western blot (WB) dilution: 1:250; anti-extracellular signal-regulated kinas (Erk1) (pT202/pY204) + Erk2 (pT185/pY187) antibody, Abcam, ab4819, rabbit polyclonal, western blot (WB) dilution: 1:1000; anti-Erk1/2 antibody, Abcam, ab17942, rabbit polyclonal, western blot (WB) dilution: 1:1000; anti-Lats antibody, Santa Cruz (Dallas, Texas, U.S.A.), sc-9388, goat polyclonal, western blot (WB) dilution: 1:200; anti-TAZ antibody, Santa Cruz, sc-48,805, rabbit polyclonal, western blot (WB) dilution: 1:500; anti-TEAD antibody, Santa Cruz, sc-134,070, rabbit polyclonal, western blot (WB) dilution: 1:1000; anti-β-actin antibody, Abcam, ab8226, mouse monoclonal, western blot (WB) dilution: 1:5000; P-MST, CST (Boston, Massachusetts, USA), #3681, rabbit polyclonal, immunohistochemistry dilution: 1:250; P-YAP, CST, # 4911, rabbit polyclonal, immunohistochemistry dilution: 1:250.

### Drug

XST (national medicine permission number Z20030017, lot number 130630) was purchased from Zhongsheng Pharmaceutical Co., Ltd. (Guangdong, China). There was 0.5 g drug per grain.

### Animals

Thirty six Male healthy, Sprague-Dawley rats (8 weeks of age, 250–300 g) were supplied by Vital River Laboratory Animal Technology Co. Ltd. (Beijing, China, Certificate no SCXK (Beijing) 2007–0001). The animals were kept at a room temperature of 22–24 °C, 40% humidity, and a 12-h daylight cycle. The rats were housed in 465 × 300 × 200 mm cages (Longdonghai Ltd., China, Type II) and provided with water and commercial rat feed ad libitum. Three rats were raised in one cage. The beddings were changed every day, and the cages were changed every week. When the experiments ended, the animals were sacrificed using intraperitoneal injections of pentobarbital (50 mg/kg). The status of rats was examined every day and the blood glucose was measured every 4 weeks.

### Induction of diabetes

Animals were fasted for 14 h before streptozotocin (STZ, Sigma Chemical Co, USA, cat# S0130) injection. STZ was dissolved in10 mM citrate buffer (pH 4.4) and intraperitoneally injected within 5 min at 65 mg/kg body weight. Age-matched control rats received equal volumes of vehicle (citrate buffer). Seven days later, blood was obtained from the tail veins for glucose analysis using a standard glucometer (One Touch Profile, Lifescan, Inc., USA). Rats with blood glucose levels higher than 16.7 mmol/L were considered to be diabetic and used for the subsequent experiments.

### Treatment schedule

After 20 weeks of diabetes induction, the diabetic rats were divided into two groups according to the glucose concentration and the body weight: diabetic (*n* = 17), XST (*n* = 8). Then the treatment with XST by intragastric gavage was started. The daily dose of XST given was 1.05 g/kg body weight, which was equivalent to approximately 7 times the amount of the dose that patients receive per day. The rats in normal group (*n* = 11) and diabetic group were fed with water at the same time. Color Doppler imaging and trypsin digest preparation were conducted after 12 weeks of XST treatment. Then the rats were sacrificed.

### Observation by Color Doppler imaging

Color Doppler imaging was used to monitor the flow velocities before the rats were killed. The blood velocities of the central retinal artery (CRA) were detected by Color Doppler (Vevo 2100, VisualSonics, Canada). The probe was placed on the opened eye following the application of sterile contact gel to minimize the force of the probe on the globe. Peak systolic velocity(PSV), end-diastolic velocity(EDV), mean velocity(MV), resistance index(RI) and pulsatility index(PI) of the CRA were measured.

### Immunohistochemical staining

The paraffined slices were deparaffinized and dehydrated. For immunohistochemistry, after the endogenous peroxidases were removed using 0.3% hydrogen peroxidase, the primary antibody against P-MST or P-YAP antibody was added to the slices and incubated at 4 °C overnight. After three washes, the sample slices were incubated with the horseradish peroxidase-conjugated secondary antibodies; 3, 3′-diaminobenzidine (DAB) was used as the chromogen. In the end, the hematoxylin staining was performed.

### Trypsin digest method

The retina was isolated and incubated at 37 °C in digestion buffer (0.1 mol/L Tris buffer, pH 7.8), containing 3% trypsin (Amresco, USA). After 2–3 h of incubation, when the internal limiting membrane began to separate from the retina, the retina was transferred to phosphate-buffered saline (pH 7.4) at room temperature. The vascular tree was washed in distilled water to be freed of any remaining neural tissue. The preparations were set on glass slides, air dried, and stained with hematoxylin and Periodic Acid-Schiff stain (PAS) to evaluate microvascular lesions. The acellular capillaries of the retina were analyzed. The number of endothelial cells and pericytes were counted, and the ratio was calculated. The endothelial cells were identified as elliptical and oriented along the circumference of the capillary, and the pericytes were defined as round in shape and abutting the outer portion of the capillary wall.

### Quantitative real-time PCR

Total RNA was extracted from frozen retina tissues. RNA concentration was determined by spectrophotometer (Nanodrop 2000, Thermo, USA). Complementary DNA was synthesized using a reverse transcription reagent kit (Roche, USA). Primers were ordered from Shanghai Shenggong Co Ltd. Amplification and quantitation were performed by real-time PCR (ABI7500, USA), and β-actin served as the control. The primers for VEGF and β-actin were as followed: VEGF primer (forward 5′-3′): CAGAAGGGGAGCAGAAAGCC, reverse (5′-3′): AATGTTCAGCCCCAACCAAGA; β-actin primer (forward 5′-3′): GCAGGAGTACGATGAGTCCG, reverse (5′-3′): ACGCAGCTCAGTAACAGTCC.

### Western blot analyses

Retinas were homogenized in RIPA buffer (Pulilai, China) containing protease inhibitors. The lysate was centrifuged, and the supernatant was collected. Protein content was assayed using the BCA protein assay (Thermo, USA). The tissue lysate, which contained 60 μg of protein, was separated on 12% SDS-polyacrylamide gels and was transferred onto polyvinylidene fluoride membranes. The membranes were blocked for 1.5 h at room temperature in 5% nonfat dried milk with TBST (Pulilai, China) and then incubated with a primary antibody overnight at 4 °C. The membranes were washed and incubated with a secondary antibody at a dilution of 1:5000 for 1 h. Finally, the membranes were washed in triplicate with TBST and developed using enhanced chemiluminescence (GE, USA). The bands on the film were measured, and density measurements were normalized to β-actin readings.

### Statistical analysis

The Shapiro-Wilk test was applied to verify the normality of the distributions. A two-way analysis of variance (ANOVA) was used to verify the differences between the normal distributions, and the Kruskal-Wallis test was used to assess differences between nonparametric distributions. For normal distributions, the results were expressed as the means ± S.D., and the differences were considered significant when the probability of a Type I error was lower than 5% (*p* < 0.05).

## Results

### Blood glucose and body weight

**Fig. 1:**
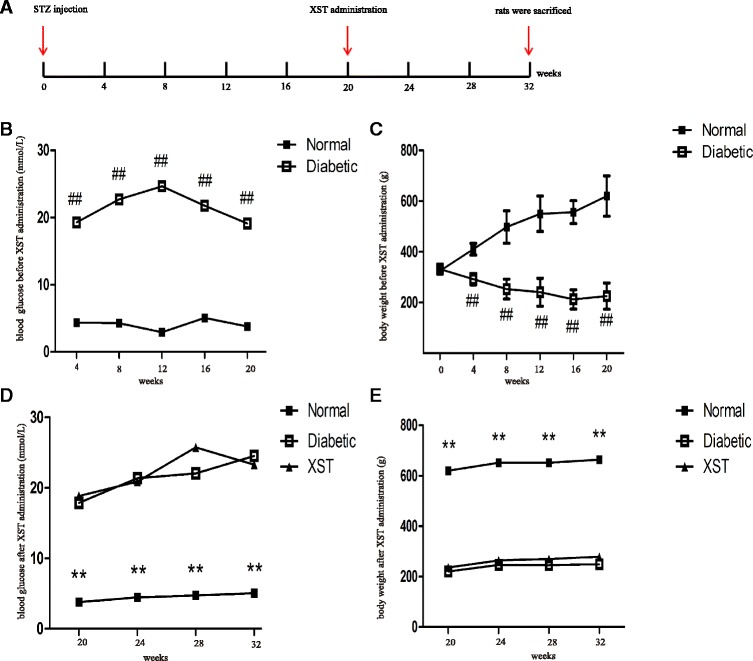
The blood glucose and body weight of rats. Diabetic rats were induced by streptozocin. The normal control rats received citrate buffer. The blood glucose and body weight were measured every 4 weeks. 20 weeks later, the rats whose blood glucose was higher than 16.7 mmol/L were divided into diabetic group and XST group. Then XST administration started and continued for 12 weeks. **a** Time schedule. **b**–**c** The blood glucose and body weight before XST administration. ##: *P* < 0.01, compared to Normal. **d**–**e** The blood glucose and body weight after XST administration. There was no significant difference between diabetic group and XST group. **: *P* < 0.01, compared to Diabetic

Blood glucose and body weight were measured every 4 weeks throughout the 32-week period. Diabetic rats maintained a higher blood glucose (19.1 mmol/L ~ 26.2 mmol/L), a 4–8-fold increase compared with the age-matched control rats (vehicle treated; *P* < 0.001), and lower body weight (*P* < 0.001) throughout the experiment period. In contrast to the diabetic group, XST had no effect on blood glucose and body weight in the controls (Fig. 1c, d; *P* > 0.05). No side- effects of XST were observed.

### Blood flow in the CRA

**Fig. 2:**
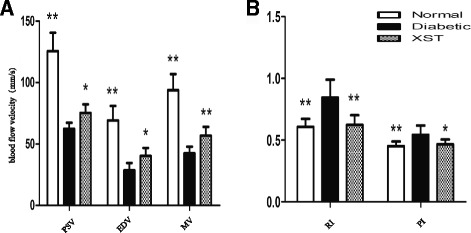
The effect of XST on blood flow in CRA of rats. At the end of the treatment, rats were anesthetized and blood flow in CRA was measured by color doppler imaging. XST had a remarkable increase in PSV, EDV and MV and lower levels of PI and RI. Data were represented as mean ± S.D. (*n* = 6–10). *: *P* < 0.05, **: *P* < 0.01, compared to Diabetic. CRA, central retinal artery; PSV, peak systolic velocity; EDV, end-diastolic velocity; MV, mean velocity; RI, resistance index; PI, pulsatility index. (**a**) PSV, EDV and MV of CRA. (**b**) RI and PI of CRA

Color Doppler imaging is a noninvasive, reproducible, and easily applied technique, and it has been shown to be useful in evaluating hemodynamic changes in several orbital and retinal vascular diseases [18]. It has been reported that blood flow velocity in the CRA was decreased in rats with DR [14]. At the end of the present experiment, blood flow in the CRA was evaluated, and similar results were observed (Fig. 2). Diabetic rats exhibited a significant reduction in blood flow and an increase in PI and RI in the CRA compared with the control rats **(** *P* < 0.05**).** PSV, EDV and MV were markedly increased in the XST group compared with the diabetic group (*P* < 0.05, Fig. 2a). XST reduced the RI and PI remarkably (*P* < 0.05, Fig. 2b).

### Retinal vascular histopathology

**Fig. 3:**
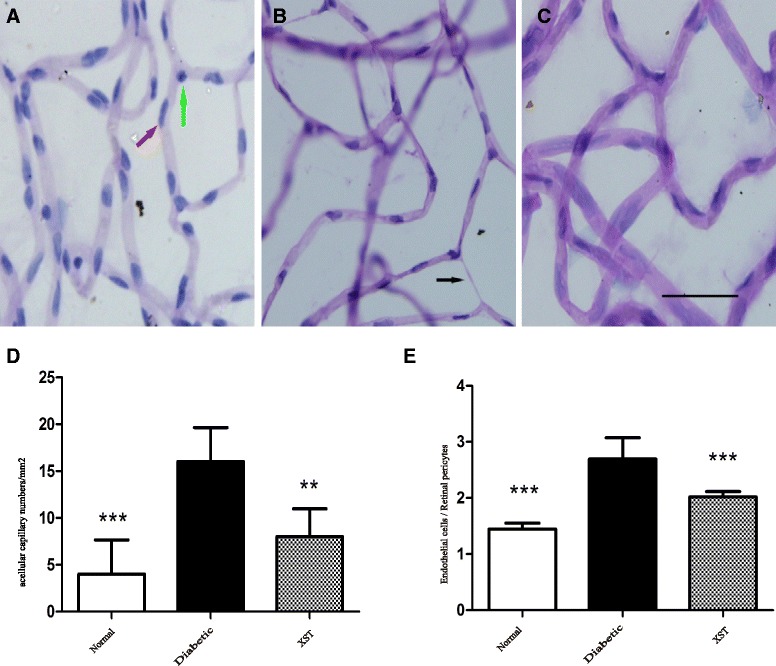
The effect of XST on morphology of rat retinas. The retinas were fixed in formalin for trypsin digestion. **a**–**c** The vasculature in midretina. Scale bar: 50 μm. The images showed the acellular capillaries (*black arrow*), endothelial cells (*green arrows*) and pericytes(*red arrow*). **d** The acellular capillaries were counted and expressed as the total number/mm of retina area. There was a remarkable decrease in cellular capillaries in XST group. **e** The endothelial cells and pericytes were identified and counted. Then the ratio of endothelial cells number /pericytes number was calculated. XST administration improved the ratio of endothelial cells number /pericytes number. Data were represented as mean ± S.D. (*n* = 3–5).*: *P* < 0.05, **: *P* < 0.01, ***: *P* < 0.001, compared to Diabetic

Previous studies in animal models have indicated that the ratio of endothelial cells to pericytes increased significantly [19]. When the XST administration ended, the retinas were removed for trypsin digestion. The results showed that acellular capillaries and the ratio of endothelial cells to pericytes in diabetic rat retinas increased compared with normal control rats (*P* < 0.001, Fig. 3). Additionally, XST reduced acellular capillaries and the ratio of endothelial cells to pericytes (*P* < 0.001, Fig. 3). These findings suggest that a microvasculature lesion developed in the diabetic rat retinas and that XST inhibited the pathological changes in diabetic retinopathy.

### Hippo pathway changes

**Fig. 4:**
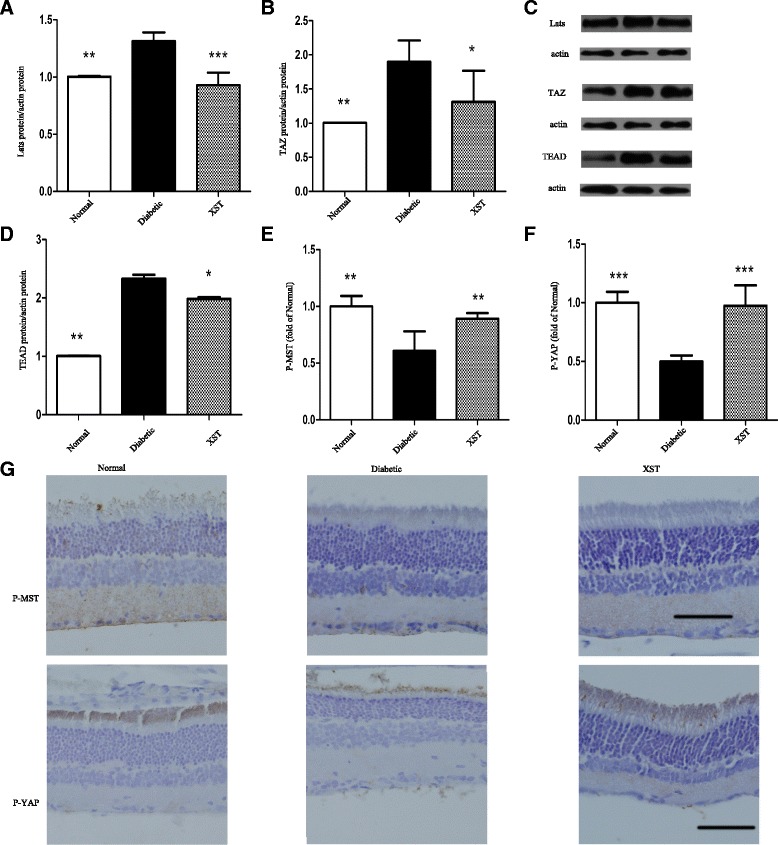
The effect of XST on protein levels of Hippo pathway. **a**–**d** the protein levels of Lats, TAZ and TEAD were evaluated by WB. XST decreased levels of Lats, TAZ and TEAD protein. **e**–**g** Distribution and expression of P-MST and P-YAP were measured using IHC. P-MST was mainly observed in inner plexiform layer and small amount was expressed in outer nuclear layer and photoreceptor layer. P-YAP was clearly seen photoreceptor layer. An elevation of P-MST and P-YAP were observed in XST group. Data were represented as mean ± S.D. (*n* = 3–4).*: *P* < 0.05, **: *P* < 0.01, ***: *P* < 0.001, compared to Diabetic

The protein levels of the core members of the Hippo pathway were determined by western blot and immunohistochemistry. The western blot results showed that the Lats protein level was increased in the retinas of diabetic rats compared with those of normal rats (*P* < 0.01, Fig. 4). Diabetic rats also exhibited induced TAZ protein expression (*P* < 0.01, Fig. 4) accompanied by an increase in TEAD protein levels (*P* < 0.01, Fig. 4).

As expected, the XST group had markedly lower levels of Lats and TAZ compared with the diabetic rats (*P* < 0.001 or *P* < 0.05, Fig. 4). Additionally, rat retinas displayed a measureable increase in the TEAD levels in response to XST administration (*P* < 0.01, Fig. 4).

Immunohistochemistry was performed to evaluate the distribution and expression levels of P-MST and P-YAP in rat retinas. As shown in Fig. 4, P-MST was mainly observed in the inner plexiform layer (IPL), and a small amount was expressed in the ONL and PL. P-YAP was clearly seen in the photoreceptor layer. In the diabetic group, P-MST and P-YAP displayed a significant decrease in the retinas compared with the normal group (*P* < 0.01 or *P* < 0.001, Fig. 4); however, rats treated with XST presented a higher expression of P-MST and P-YAP in their retinas (*P* < 0.01 or *P* < 0.001, Fig. 4).

### VEGF levels in retina

**Fig. 5:**
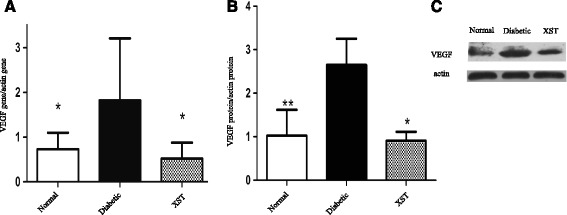
The effect of XST on gene and expression levels of VEGF. **a** The gene level of VEGF was determined by PCR. XST lead to a reduction of VEGF gene level. **b**–**c** The protein level of VEGF was evaluated by WB. XST displayed a decrease of VEGF protein level. Data were represented as mean ± S.D. (*n* = 3–4).*: *P* < 0.05, **: *P* < 0.01, compared to Diabetic

Previous evidence has shown that VEGF is one of the most important mediators for DR; therefore, the VEGF gene and protein levels were examined in the present study. PCR and western blot analysis showed that the VEGF gene and protein levels increased significantly in the retinas of the diabetic group compared with those of the normal control group (*P* < 0.01, Fig. 5). Treatment with XST decreased VEGF gene and protein expression (*P* < 0.01, Fig. 5).

### P-ERK1/2 levels in retina

**Fig. 6:**
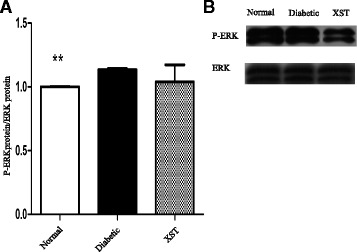
The effect of XST on protein levels P-ERK and ERK. **a** The ratio of P-ERK/ERK was compared. **b** The images of WB results. XST decreased the ratio of P-ERK/ERK. Data were represented as mean ± S.D. (*n* = 3–4). **: *P* < 0.01, compared to Diabetic

P-ERK1/2 and ERK1/2 protein expression analysis was performed using western blot, and the ratio of P-ERK/ERK was calculated. Increased P-ERK1/2 was observed in the diabetic group compared to the normal group (*P* < 0.01, Fig. 6). Compared with diabetic group, the level of P-ERK1/2 in the retinas of rats was elevated in XST group (*P* > 0.05, Fig. 6).

## Discussion

In this study, the results showed that the Hippo pathway became dysregulated in the retinas of diabetic rats, and XST restored the protein levels of this signaling pathway. Therefore, we describe here a novel molecular mechanism by which the Hippo pathway may be involved and play an important role in DR. Additionally the Hippo pathway has been implicated in underlying the curative effect of XST.

Previous results indicated that the Hippo pathway mediates angiogenesis, which is related to the damages within the retina in DR. In addition, VEGF has been implicated as one of the most important cytokines with angiogenic and mitogenic actions, which has great effects on DR. It has been confirmed that the overexpression of TEAD increased VEGF promoter activity and VEGF expression in endothelial cells [20]. Additionally, silencing YAP inhibited the expression of VEGF [21]. Taken together, these reports suggest that the Hippo pathway is involved in cell proliferation and angiogenesis by regulating VEGF. Although not all of the participants in the crosstalk between the Hippo pathway and VEGF are fully understood, ERK may function as an intermediary. YAP, the reporter of the Hippo pathway, is able to induce ERK phosphorylation [22], and the ERK pathway has been shown to increase VEGF mRNA stability [23] and promote VEGF expression [24]. Previous reports have suggested that the Hippo pathway activates ERK, which modulates VEGF and facilitates angiogenesis.

Our findings that MST, Lats, YAP, TAZ and TEAD were all altered in the retinas of rats with DR led us to conclude that the Hippo pathway may be the underlying factor in the process of DR. Additionally, VEGF and P-ERK were elevated in diabetic rats, which implies that P-ERK-VEGF is the downstream target of the Hippo pathway in DR.

Although the way that the Hippo pathway participates in DR has not been examined, several upstream regulators of the Hippo pathway, such as GPCR, SCRIB and cadherin [5], are suspected to regulate vascular cell proliferation, migration [25, 26] or insulin secretion [27]. Thus, Hippo pathway activity is tightly coupled to angiogenesis and glucose metabolism. Angiogenesis and hyperglycemia are fundamental features of DR and might thus underlie the deregulated Hippo pathway activity in DR.

In the current study, XST affected the protein levels of MST, Lats, YAP, TAZ and TEAD. These results suggest that the Hippo pathway could be a therapeutic target for DR. XST is composed of many major active constituents, including tanshinone, saponins, harpagoside, astragaloside and flavonoids. Tanshinone-IIA and cryptotanshinone [28], Ginsenoside Rd. [29], notoginsenoside R1 [30], Aucubin [31] are responsible for the regulation of VEGF or P-ERK. The VEGF, P-ERK and Hippo signaling pathways could interact and contribute to the pathological process, so it has been proposed that some of the active constituents isolated from XST can collaborate to control retinal endothelial cell proliferation or migration and inhibit angiogenesis.

## Conclusions

**Fig. 7:**
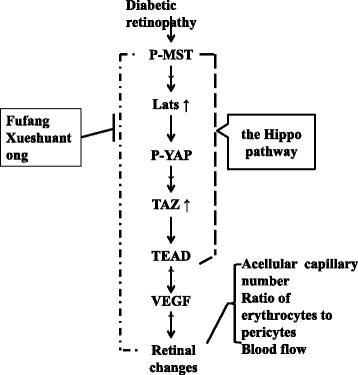
the Hippo pathway is involved in diabetic retinopathy, which is inhibited by Fufang Xueshuantong

In conclusion (Fig. 7), this study documents an important role for the Hippo pathway in the process of DR and offers a potential drug target for the management of DR. However in later experiments, maybe it is necessary to confirm the importance of Hippo pathway to DR by knockdown technology. Furthermore, this study demonstrates that XST exhibits effects on the Hippo pathway in rats with DR and provides molecular mechanisms underlying the use of XST for the treatment of DR.

## Additional file

Additional file 1: Data of the expreiment. (XLS 39 kb)

## Abbreviations

CRA: Central retinal artery
DAB: 3, 3′-diaminobenzidine
DR: Diabetic retinopathy
EDV: End-diastolic velocity, EDV
ERK: Extracellular signal-regulated kinas
hsav1: Salvador1
ICAM-1: Intercellular cell adhesion molecule-1
iNOS: Inducible nitric oxide synthase
Lats: Large tumor suppressor homolog
MDA: Malondialdehyde
Mob1: MOB kinase activator1
MST: mammalian sterile 20-like
MV: Mean velocity, MV
PAS: Periodic acid-schiff stain
PEDF: Pigment epithelium-derived factor
PI: Pulsatility index, PI
PSV: Peak systolic velocity
RI: Resistance index, RI
SOD: Superoxide dismutase
TAZ: Transcriptional co-activator with PDZ binding motif
TEAD: TEA domain family members
VEGF: Vascular endothelial growth factor
WB: Western blot
XST: Fufang Xuesuhangtong
YAP: Yes-associated protein
